# Editorial: Deregulated signaling pathways in inflammation and cancer

**DOI:** 10.3389/fcell.2024.1459926

**Published:** 2024-07-19

**Authors:** Parameswaran Ramakrishnan

**Affiliations:** ^1^ Department of Pathology, Cleveland, OH, United States; ^2^ The Case Comprehensive Cancer Center, Cleveland, OH, United States; ^3^ Department of Biochemistry, Cleveland, OH, United States; ^4^ University Hospitals-Cleveland Medical Center, School of Medicine, Case Western Reserve University, Cleveland, OH, United States; ^5^ Louis Stokes Veterans Affairs Medical Center, Cleveland, OH, United States

**Keywords:** cell signaling, inflammation, cancer, inflammatory diseaes, chemoresistance

Signaling pathways govern how cells survive, proliferate, communicate and respond to their environment. Alterations in signaling pathways can lead to dysregulated immune and inflammatory responses or uncontrolled cell growth. Unresolved inflammation is an underlying cause of many diseases including cancer (Greten and Grivennikov, 2019; Turizo-Smith et al., 2024). In both inflammation and cancer, dysregulation of one or more signaling pathways involving altered transcription, translation, posttranscriptional and posttranslational regulations, mutations affecting protein and nucleic acid functions as well as epigenetic modifications are associated with the pathology of the disease. Hence, understanding deregulated signaling mechanisms that leads to the disease condition is necessary to develop targeted therapeutics with minimal off-target effects. Deciphering deregulated signaling pathways will contribute to the development of early intervention therapies, identification of altered gene expression that may serve as potential biomarkers and reveal novel targets for combination therapy.

This Research Topic comprises a diverse set of six comprehensive reviews and an original article covering selected signaling pathways involved in inflammation and cancer. The original research article by Hernandez-Cano et al. presents key findings on the role of C3G (RapGEF1) in platelet-dependent angiogenesis and tumor metastasis. This study suggests that C3G expression should be tightly regulated in platelets and megakaryocytes to prevent megakaryocytosis, thrombocytosis and myeloproliferative disorders. Further, suggesting a global signaling role of C3G, preliminary experiments presented show that absence of C3G in platelets compromised proteasomal and lysosomal degradation, which may allow the sustained expression of many proteins involved in inflammation and cancer.

Each of the review article has diligently compiled information from over hundred other articles to provide insightful updates on the respective subject areas discussed. The reviews by Hu et al. and Deng et al. focus on liver and intestinal inflammation, respectively. Hu et al. have summarized the signaling roles of TRIF protein, a crucial adaptor molecule downstream of toll-like receptors 3 (TLR3) and 4 (TLR4). Notably, specific roles TRIF in multiple signaling pathways emanating from TLR3 and TLR4 are discussed along with an in-depth discussion on the role of TRIF in various hepatic inflammatory diseases as well as liver cancer and fibrosis. The detailed description of biochemical mechanisms involving TRIF and its link to pathologies successfully presents the potential to target TRIF and associated pathways to treat liver diseases.

Deng et al. reviewed the intersection of two very important signaling pathways, ferroptosis and Hippo pathway with specific focus on intestinal diseases and intestinal cancer. Elaborate discussions are included on the roles of Hippo pathway in metabolism, immune response, mucosal barrier, mechanical stress and intestinal tumors. Similarly, the roles of ferroptosis, a regulated cell death resulting from iron accumulation (Chen et al., 2024), in inflammation, immune response, cancer, metabolism and mechanotransduction are also discussed. Interestingly, attention has been drawn to the overlapping roles of Hippo pathway and ferroptosis in intestinal diseases, by specifically calling out potential mediators that bridge these pathways. These include GPX4, ROS, gut microbiota, p53 and YAP among several others. In conclusion, the authors have correctly acknowledged that current research on the importance of Hippo pathway and ferroptosis is confined to intestine and future studies are warranted to expand this to diseases of other organs as well.

The reviews by Wei et al. and Le focus on inflammation and cancer. Wei et al. elaborated the mechanisms involving two reciprocal regulators of inflammation: the anti-inflammatory histone deacetylase SIRT1 and the pro-inflammatory HMGB1. The authors have compendiously presented the functions of SIRT1 and HMGB1 in various inflammatory conditions and cancers alluding to the cross talk between these two molecules, offering a foundational review that may aid future studies and therapeutic explorations of these molecules.

Le has presented a detailed review of catalytic-dependent and -independent roles of the MAPK protein ERK5 and elaborated on its role in multiple cancers and cancer associated inflammation. Description of the various signaling pathways that converges on ERK5, itemized presentation of its key substrates and functions, and posttranslational modifications of ERK5 presents valuable insights on ERK5 associated signaling mechanisms. A critical view on both pro- and anti-inflammatory roles of ERK5 is also presented that should be considered to avoid undesirable outcomes while pursuing disease-dependent therapeutic development.

Ozcan has elegantly reviewed the role of HIF-1α in stemness of gastric cancer cells and their resistance to chemotherapy. Targeting HIF-1α mediated signaling appears to be a potential approach to block reprogramming of cancer cells to stem cells and prevent chemoresistance, however, this review call attention to the need for more knowledge on the HIF-1α−dependent molecular mechanisms in these processes. Author has critically evaluated the known mechanisms and compiled critical details of several available compounds targeting HIF-1α including summary of their clinical trial status, providing a near complete picture of the status of this field.

Rana et al. discussed drug resistance in multiple myeloma, a major challenge that hinder the successful outcomes of current treatments. They reviewed the roles of TGF*-*β in tumor microenvironment in suppressing host immunity and its tumor intrinsic role in chemoresistance. They have also included highly relevant discussion on the possibilities of targeting TGF*-*β in multiple myeloma to achieve better outcomes when using immunomodulatory drugs, TCR-engineered cells or CAR-T cells, and bi-specific T cell engagers as anti-tumor therapies.

Overall, the extent to which the articles included in this topic delved into the literature is appreciable and they cover research areas that are relatively less explored providing a wealth of timely information that is expected to facilitate future research.
